# Cause-specific excess mortality after hip fracture: the Norwegian Epidemiologic Osteoporosis Studies (NOREPOS)

**DOI:** 10.1186/s12877-023-03910-5

**Published:** 2023-03-31

**Authors:** Kristin Holvik, Christian Lycke Ellingsen, Siri Marie Solbakken, Trine Elisabeth Finnes, Ove Talsnes, Guri Grimnes, Grethe S. Tell, Anne-Johanne Søgaard, Haakon E. Meyer

**Affiliations:** 1 Department of Physical Health and Ageing, Norwegian Institute of Public Health, P. O. Box 222, Skøyen, Oslo, 0213 Norway; 2 Department of Pathology, Stavanger University Hospital, Stavanger, Norway; 3 Department of Global Public Health and Primary Care, University of Bergen, Bergen, Norway; 4 Department of Endocrinology, Innlandet Hospital Trust, Hamar, Norway; 5 Department of Endocrinology, Oslo University Hospital, Oslo, Norway; 6 Department of Orthopedics, Innlandet Hospital Trust, Elverum, Norway; 7 Department of Clinical Medicine, UiT The Arctic University of Norway, Tromsø, Norway; 8 Division of Internal Medicine, University Hospital of North Norway, Tromsø, Norway; 9 Department of Community Medicine and Global Health, University of Oslo, Oslo, Norway

**Keywords:** Hip fracture, Causes of death, Excess mortality, Registry-based epidemiology, Norway

## Abstract

**Background:**

Information on cause of death may help appraise the degree to which the high excess mortality after hip fracture reflects pre-existing comorbidities or the injury itself. We aimed to describe causes of death and cause-specific excess mortality through the first year after hip fracture.

**Methods:**

For studying the distribution of causes of death by time after hip fracture, we calculated age-adjusted cause-specific mortality at 1, 3, 6 and 12 months in patients hospitalized with hip fracture in Norway 1999–2016. Underlying causes of death were obtained from the Norwegian Cause of Death Registry and grouped by the European Shortlist for Causes of Death. For estimating excess mortality, we performed flexible parametric survival analyses comparing mortality hazard in patients with hip fracture (2002–2017) with that of age- and sex matched controls drawn from the Population and Housing Census 2001.

**Results:**

Of 146,132 Norwegians with a first hip fracture, a total of 35,498 (24.3%) died within one year. By 30 days post-fracture, external causes (mainly the fall causing the fracture) were the underlying cause for 53.8% of deaths, followed by circulatory diseases (19.8%), neoplasms (9.4%), respiratory diseases (5.7%), mental and behavioural disorders (2.0%) and diseases of the nervous system (1.3%). By one-year post-fracture, external causes and circulatory diseases together accounted for approximately half of deaths (26.1% and 27.0%, respectively). In the period 2002–2017, cause-specific one-year relative mortality hazard in hip fracture patients vs. population controls ranged from 1.5 for circulatory diseases to 2.5 for diseases of the nervous system in women, and correspondingly, from 2.4 to 5.3 in men.

**Conclusions:**

Hip fractures entail high excess mortality from all major causes of death. However, the traumatic injury of a hip fracture is the most frequently reported underlying cause of death among older patients who survive less than one year after their fracture.

## Background

Hip fractures are associated with substantial excess mortality, which is particularly high shortly after the event, but persists for many years after the fracture [1–3]. A hip fracture, usually caused by a fall, is a serious injury particularly affecting older adults. Acutely after a hip fracture there is high risk of complications and health may deteriorate rapidly. Meanwhile, many patients are characterised by pre-fracture frailty, functional impairment, and multimorbidity [4]. To help target resources for prevention and care, we should elucidate the degree to which the excess mortality after hip fracture reflects comorbid diagnoses versus the trauma of the injury itself. To this end, the causes of death recorded on the death certificate provide useful information. Using nationwide registry data from Norway over two decades, we aimed to (1): describe the distribution of causes of death after hip fracture by time since the fracture, and (2): quantify the cause-specific excess one-year mortality after hip fracture compared with the background population.

## Methods

### Study population

For the first aim, to examine the distribution of causes of death through the first year after hip fracture, all patients 50 years and older who had a first incident hip fracture treated in hospitals in Norway 1999–2016 comprised the study population (see section *Exposure*). For the second aim, to examine cause-specific excess mortality after hip fracture, the study population was defined as all inhabitants identified in the Norwegian Population and Housing Census carried out in November 2001 who were aged 50 years and older in 2001, had not had a hip fracture during 1994 through 2001, and were alive and resided in Norway on January 1^st^ 2002. These individuals were followed from January 1^st^ 2002 through December 31^st^ 2017 with regard to incident hip fractures and deaths.

### Exposure: Hip fractures

In Norway, all patients with hip fracture are admitted to a hospital. Hip fractures treated in hospitals from 1994 onwards were available in the Norwegian Epidemiologic Osteoporosis Studies (NOREPOS) hip fracture database (NORHip) [5–8]. This database contains inpatient data obtained from hospitals’ patient administrative systems (1994–2007) and from the Norwegian Patient Registry (2008 onwards). We identified incident hip fractures and discriminated between individuals’ first and second hip fracture during the period by applying an algorithm that considered the combination of hip fracture diagnosis codes (International Classification of Diseases, 10^th^ edition (ICD-10): S72.0, S72.1 and S72.2), surgical procedure codes, additional diagnosis codes and time between hospitalizations. The database has been validated [9]. For the current analysis, a five-year washout period (1994–1998) was used to reliably identify the individual’s first (incident) hip fracture. The information from NORHip was linked to other registry data using the unique 11-digit personal identification number assigned to every resident in Norway.

### Outcome: Cause-specific mortality

Dates and causes of all deaths 1999–2017 were available from the Norwegian Cause of Death Registry. This registry is managed by the Norwegian Institute of Public Health and contains digitized cause of death data by age, sex, place of death, and place of residence [10]. Deaths are reported by physicians through completing mandatory death certificates. Autopsy reports and notifications of deaths from other agencies comprise additional sources of information. Since 1996, diagnoses for immediate, underlying and contributing causes of death are coded according to ICD-10. The underlying cause of death is defined as the disease or injury that initiated the train of morbid events leading directly to death, or the circumstances of the accident or violence that produced the fatal injury [11, 12]. ICD coding rules determine the logical order of immediate, underlying, and contributing causes of death. Deaths caused by injuries, including fractures, must be assigned an external cause (ICD-10 codes V01-Y98) as the underlying cause of death. Hip fractures usually occur from a fall, often from standing height or less. From 2005 onwards, the selection of the appropriate underlying cause of death, based on the diagnoses codes on the death certificate and the ICD guidelines for their expected interrelationships, was semi-automated using the Automated Classification of Medical Entities (ACME) system. ACME was developed to help standardise coding practices and improve comparability of cause of death statistics [13–15]. In Norway ACME has been incorporated into the Iris software [16]. Before 2005, guidelines instructed coding of deaths caused by hip fractures as unspecified falls (ICD-10: W19) if no external cause had been recorded. After implementing ACME, ICD-10 code X59.0 ‘Exposure to unspecified factor causing fracture’ is assigned to deaths caused by hip fractures if the external cause is missing. Therefore, both deaths from ICD-10 codes X59.0 and W19 that occur after a hip fracture are interpreted to be caused by the fracture event. For the purpose of cause of death statistics, the underlying causes of death are grouped according to the European Shortlist for Causes of Death as established by Eurostat [17]. This shortlist covers 17 broad categories of diagnoses, principally grouped by ICD chapters, which are further divided into 65 subcategories.

### Statistical analyses

Data preparation and statistical analyses were performed in R for Windows, version 4.1.1 [18]. Survival analyses (see below) were performed in Stata SE version 17 [19].

#### Mortality

To describe causes of death by time after hip fracture (aim 1) we used data from hospital stays with hip fracture diagnoses 1999 through 2016 and causes of death 1999 through 2017. We calculated the cumulative incidence of death (hereafter referred to as mortality risk; %) within 30 days, 3 months (90 days), 6 months (180 days), and 1 year (365 days) after the fracture date. In addition to describing the most common single leading diagnosis codes for underlying causes of death, the number of deaths, crude and age-adjusted mortality risks (%) were calculated for all-cause mortality and for the six most common cause of death categories based on the European Shortlist. These categories included: Neoplasms (ICD-10: C00-D48), mental and behavioural disorders (ICD-10: F01-F99), diseases of the nervous system and the sense organs (ICD-10: G00-H95), diseases of the circulatory system (ICD-10: I00-I99), diseases of the respiratory system (ICD-10: J00-J99), and external causes of morbidity and mortality (ICD-10: V01-Y89). The remaining categories each contributed less than 3% of one-year deaths and were combined into the category ‘other causes’. This category thus included deaths caused by digestive diseases, endocrine, nutritional and metabolic diseases, genitourinary diseases, infectious and parasitic diseases, musculoskeletal diseases, immunologic disorders, diseases of the skin or subcutaneous tissue, congenital malformations, and ill-defined/unspecified causes. Age- and sex-adjusted mortality risk estimates were obtained by regressing cause-specific deaths on age and sex using the function 'glm' with 'family = binomial' and 'link = logit', available in the package 'stats' in R [18].

#### Excess mortality

For examining cause-specific excess mortality the first year after hip fracture (aim 2), a matched cohort was prepared by exposure density sampling following the procedure proposed by Ohneberg [20, 21], using the 'ccwc' function of the 'Epi' package in R [22]. Briefly, on the date of each incident hip fracture that occurred during 2002–2017, three controls matched to the patient on sex and year of birth were drawn randomly from the study population. Eligibility was conditioned on being alive, residing in Norway and free of hip fracture on the patient’s hip fracture date (defined as the index date). ‘Free of hip fracture’ indicates not having a hip fracture identified in the NOREPOS hip fracture database in the period from 1 January 1994 until the index date. Statistical analyses were performed separately for the outcomes described above, namely all-cause deaths, the six major cause of death categories and ‘other causes’. For each outcome, patients and controls were followed from the index date until the date of cause-specific death or censoring due to death of any other cause (except when all-cause deaths constituted the outcome), emigration, a second hip fracture (in patients), a first hip fracture (in controls) or end of follow-up after 365 days, whichever occurred first. In accordance with previous knowledge [3], statistical tests and plots of Schoenfeld residuals [23] showed a clear time dependency for excess mortality after hip fracture. Therefore, we estimated both overall one-year and time-varying hazard ratios (HR) with 95 percent confidence intervals (95% CI) for all-cause and cause-specific deaths in flexible parametric survival models, using the function 'stpm2' in Stata [24]. Overall HRs were age-adjusted and presented separately by sex, while time-dependent HRs were estimated in men and women combined and included adjustment for age, sex and year of birth [25].

## Results

### Hip fractures and deaths

During 1999–2016, a total of 146,132 Norwegians aged 50 years and older suffered an incident hip fracture. Among these there were 44,239 (30%) men and 101,893 (70%) women. Median age at first hip fracture was 81 years (interquartile range 72–86 years) in men and 83 years (interquartile range 77–88 years) in women. A total of 20,694 hip fractures occurred in people aged 50–69 years (comprising 14%), 66,488 (46%) in those 70–84 years and 58,950 (40%) in people aged 85 years and older. The number (%) of deaths were 11,322 (7.7%) within 30 days, 20,256 (13.9%) within 3 months, 26,772 (18.3%) within 6 months, and 35,498 (24.3%) within one year after the hip fracture (Table 1).

**Table 1:** Mortality by underlying causes of death and time after hip fracture, number and % ^1^

	**30 days**			**3 months** **(90 days)**			**6 months** **(180 days)**			**1 year** **(365 days)**		
**Cause of death**	**n deaths**	**Crude %**	**Age adjusted % **^**2**^	**n deaths**	**Crude %**	**Age adjusted %**^**2**^	**n deaths**	**Crude %**	**Age adjusted % **^**2**^	**n deaths**	**Crude %**	**Age adjusted % **^**2**^
**Men (*n* = 44,239)**												
** Total deaths**	**5,089**	**11.5**	**10.1**	**8,565**	**19.4**	**19.6**	**10,969**	**24.8**	**27.8**	**14,160**	**32.0**	**41.4**
External causes	2,707	6.1	4.4	3,431	7.8	5.6	3,619	8.2	6.0	3,787	8.6	6.4
Circulatory diseases	918	2.1	1.6	1,755	4.0	3.1	2,445	5.5	4.6	3,392	7.7	6.8
Neoplasms	525	1.2	1.2	1,288	2.9	3.0	1,847	4.2	4.3	2,582	5.8	6.2
Respiratory diseases	377	0.9	0.7	789	1.8	1.5	1,115	2.5	2.2	1,625	3.7	3.3
Mental/behavioural	117	0.3	0.2	264	0.6	0.5	411	0.9	0.7	599	1.4	1.1
Nervous system	62	0.1	0.1	194	0.4	0.4	318	0.7	0.7	487	1.1	1.1
Other causes ^3^	383	0.9	0.7	844	1.9	1.7	1,214	2.7	2.4	1,688	3.8	3.5
**Women (*n* = 101,893)**												
** Total deaths**	**6,233**	**6.1**	**5.3**	**11,691**	**11.5**	**10.9**	**15,803**	**15.5**	**15.9**	**21,338**	**20.9**	**23.5**
External causes	3,388	3.3	2.5	4,741	4.7	3.4	5,169	5.1	3.8	5,477	5.4	4.1
Circulatory diseases	1,322	1.3	1.0	2,855	2.8	2.2	4,243	4.2	3.4	6,191	6.1	5.2
Neoplasms	544	0.5	0.5	1,405	1.4	1.4	2,114	2.1	2.1	3,067	3.0	3.0
Respiratory diseases	268	0.3	0.3	680	0.7	0.6	1,103	1.1	1.0	1,740	1.7	1.6
Mental/behavioural	110	0.1	0.1	405	0.4	0.3	683	0.7	0.5	1,150	1.1	0.9
Nervous system	86	0.1	0.1	294	0.3	0.3	483	0.5	0.5	744	0.7	0.7
Other causes ^3^	515	0.5	0.4	1,311	1.3	1.0	2,008	2.0	1.6	2,969	2.9	2.5

^1^Men and women aged 50 years and older with hip fracture in Norway 1999–2016. The Norwegian Epidemiologic Osteoporosis Studies (NOREPOS). Causes of death are grouped according to the European Shortlist for Causes of Death 2012 [17]

^2^Predicted at mean age at hip fracture (78.7 years in men and 81.6 years in women) from a logistic model (general linear model with 'family = binomial' and 'link = logit') regressing cause-specific death on age separately for men and women

^3^The category “Other causes” includes deaths within the following categories that each contributed less than 3% of total one-year deaths: Diseases of the digestive system; Diseases of the genitourinary system; Endocrine, nutritional and metabolic diseases; Infectious and parasitic diseases; Diseases of the musculoskeletal system/connective tissue; Diseases of the skin and subcutaneous tissue; Diseases of the blood and blood-forming organs and certain disorders involving the immune mechanism; Congenital malformations and chromosomal abnormalities; Symptoms, signs, ill-defined causes; Missing cause of death (two individuals only)

### Major causes of death by time after hip fracture

**Fig. 1:**
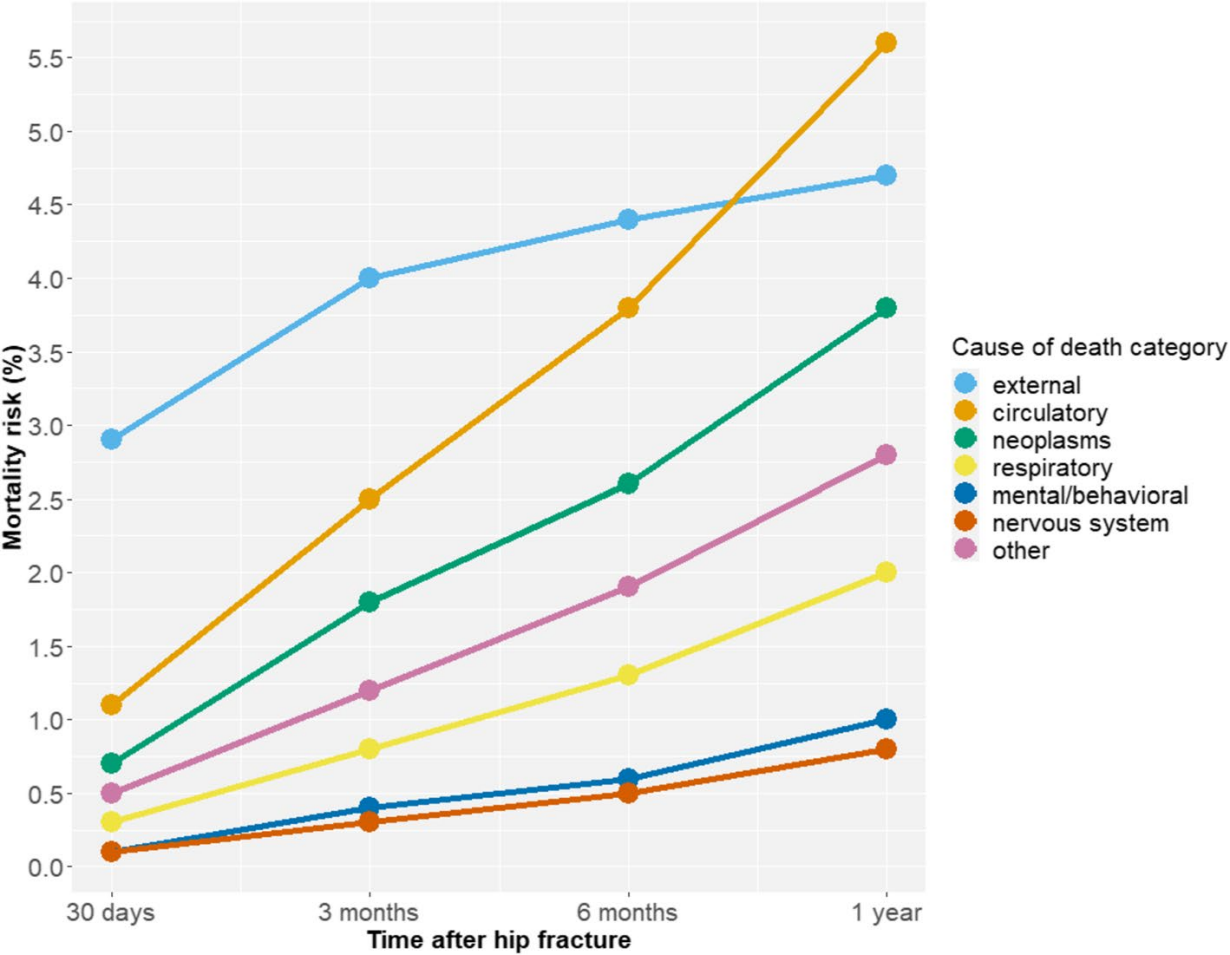
Age- and sex adjusted cause-specific mortality (%) by time after hip fracture. Norwegians 50 years and older, men and women combined (*n* = 146,132). Predicted at mean values of age at first hip fracture (80.7 years) and sex (30.3% men) from a logistic model (general linear model with ‘family = binomial’ and ‘link = logit’) regressing cause-specific death on age and sex. External: External causes of morbidity and mortality (ICD-10: V01-Y89); Circulatory: Diseases of the circulatory system (ICD-10: I00-I99); Neoplasms: Neoplasms (ICD-10: C00-D48); Respiratory: Diseases of the respiratory system (ICD-10: J00-J99); Mental/behavioral: Mental and behavioural disorders (ICD-10: F01-F99); Nervous system: Diseases of the nervous system and the sense organs (ICD-10: G00-H95); Other: Deaths caused by digestive diseases, endocrine, nutritional and metabolic diseases, genitourinary diseases, infectious and parasitic diseases, musculoskeletal diseases, immunologic disorders, diseases of the skin or subcutaneous tissue, congenital malformations, and ill-defined/unspecified causes

**Fig. 2:**
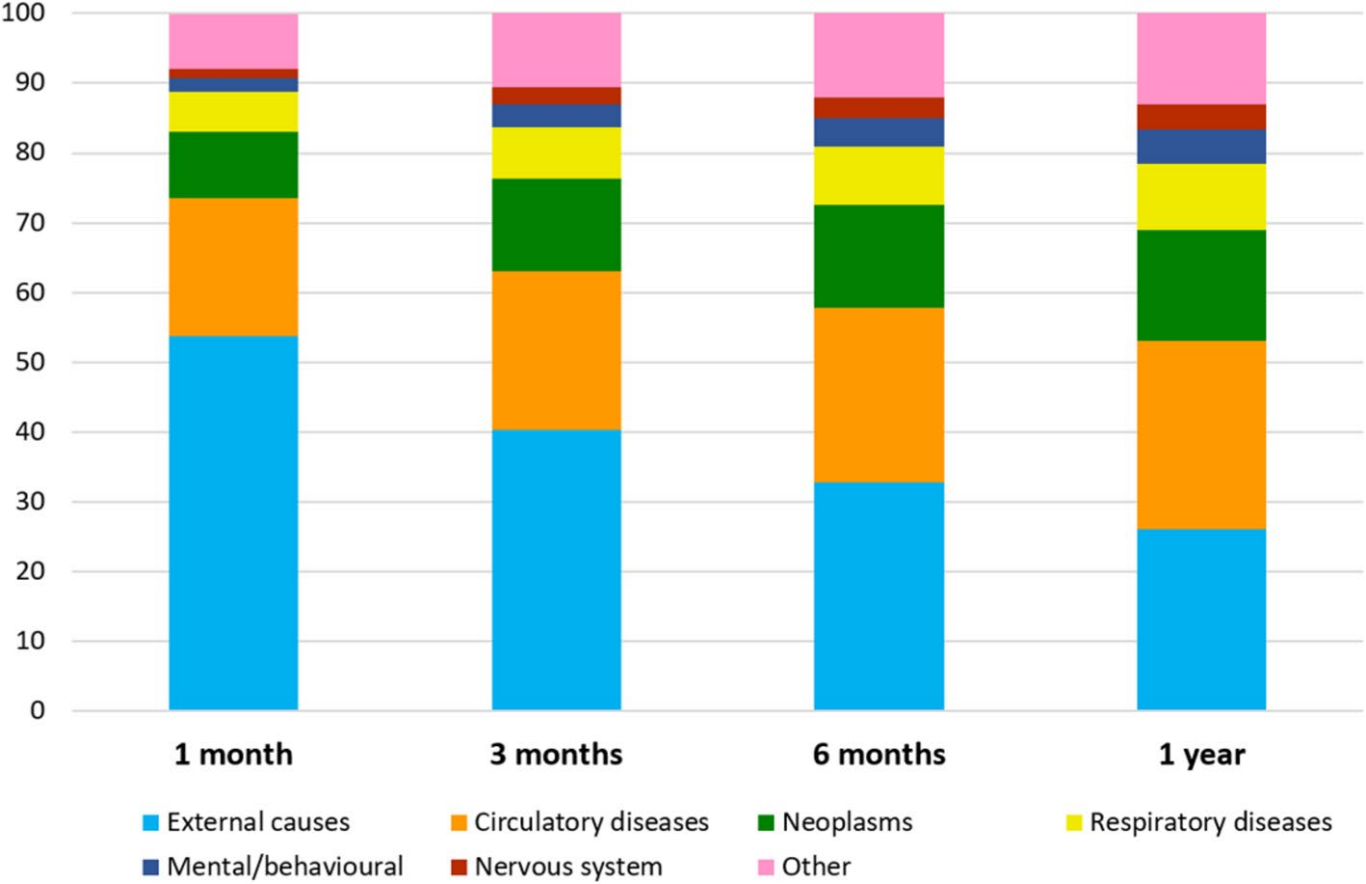
Distribution (%) of deaths after hip fracture by seven major cause of death categories. Grouped according to the European Shortlist for Causes of Death by 30 days (*n* = 11,322 deaths), 3 months (*n* = 20,256 deaths), 6 months (*n* = 26,772 deaths) and 1 year post-fracture (*n* = 35,498 deaths)

Figure 1 shows the age- and sex adjusted cause-specific mortality risk (%) by time after a patient’s first hip fracture (30 days, 3 months, 6 months and 1 year) by categories defined by the European Shortlist of Causes of Death in men and women combined. Correspondingly, Table 1 shows the numbers and proportions of deaths by time after hip fracture in each cause of death category in men and women separately. Figure 2 shows the crude percentage distribution of grouped causes of death (summing up to 100%) among those who died within one year after hip fracture. Just over half of deaths that occurred by 30 days after a hip fracture (53.8%) were reported to be caused by an external event (mainly the accident leading to hip fracture, in other words the fracture event itself). This proportion decreased with increasing time after hip fracture. Among the deaths that occurred within one year after hip fracture, 26.1% were due to external causes while a similar proportion (27.0%) were caused by circulatory diseases, most prominently acute myocardial infarction, stroke, and heart failure. These were followed by neoplasms (15.9% of one-year deaths), respiratory diseases (9.5%, predominantly pneumonia and chronic obstructive pulmonary disease), mental and behavioural disorders (4.9%, predominantly unspecified dementia) and diseases of the nervous system and the sense organs (3.5%, predominantly Alzheimer disease and Parkinson disease).

### Subsequent hip fractures

Among the 35,498 patients who died within a year after their first hip fracture, 1,078 (3%) suffered a second hip fracture between their initial hip fracture and time of death. Of these, 274 (25.4%) had an external cause, assumedly the second hip fracture injury event, registered as the underlying cause of death, while 273 (25.3%) had a circulatory disease recorded as the underlying cause of death, comparable to the proportions in all patients with a first hip fracture. Among the deaths from external causes occurring within a year after the initial hip fracture, no deaths within 30 days happened after a subsequent hip fracture, 62 (3%) of the deaths between 30 days and three months happened after a subsequent hip fracture, 88 (14%) of the deaths between three and six months happened after a subsequent hip fracture, and 124 (26%) of the deaths from external causes between six months and one year after the initial hip fracture happened after a subsequent hip fracture.

### Leading single causes of death after hip fracture

In the patients who had a hip fracture during 1999–2016 and died within the subsequent year (*n* = 35,498), the two most common single underlying cause of death diagnoses were X59.0 ‘exposure to unspecified factor causing fracture’ (*n* = 4,027) and W19 ‘unspecified fall’ (*n* = 3,786), which together accounted for 22% of the one-year deaths. These were followed by the diagnoses I21.9 ‘acute myocardial infarction, unspecified’ (*n* = 2,077; 5.9% of deaths), F03 ‘unspecified dementia’ (*n* = 1,472; 4.1% of deaths), and J18.9 ‘lobar pneumonia, unspecified’ (*n *= 1,448; 4.1% of deaths).

### Excess mortality

**Fig. 3:**
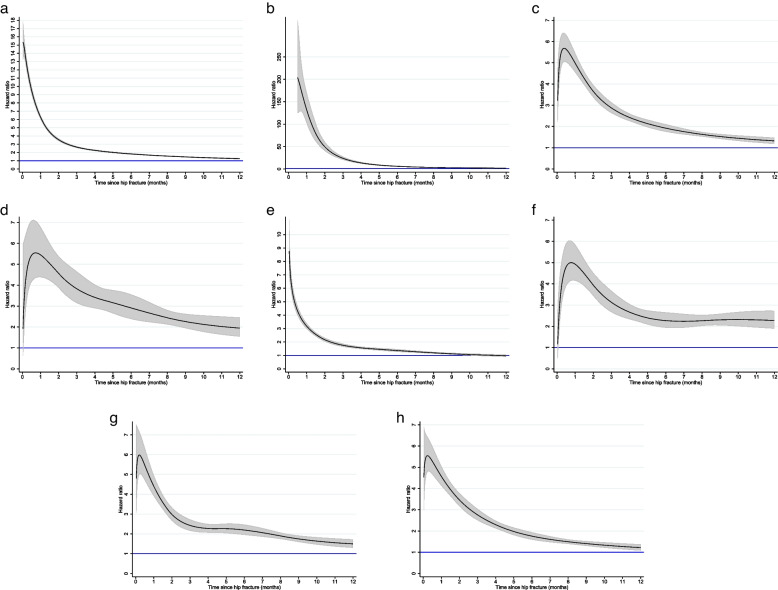
All-cause and cause-specific excess mortality (HR with 95% CI) by months after hip fracture. Panel **a**: all-cause deaths; Panel **b**: deaths from exter­nal causes; Panel **c**: deaths from neoplasms; Panel **d**: deaths from diseases of the nervous system and the sense organs; Panel **e**: deaths from circulatory diseases; Panel **f**: deaths from mental and behavioural disorders; Panel **g**: deaths from diseases of the respiratory system; Panel **h**: Deaths from other causes

Among inhabitants aged 50 years and older identified in the Norwegian Population and Housing Census 2001 who were alive and resided in Norway on January 1^st^ 2002 and had not had a previous hip fracture (based on washout back to 1994), a total of 123,130 individuals suffered a hip fracture during 2002–2017. Compared with their age- and sex-matched counterparts without hip fracture, the fracture patients had increased risk of dying from all major causes of death within the first year, and the excess mortality was higher in men than in women (Table 2). The relative risk of dying from external causes was 48-fold in male and 34-fold in female hip fracture patients. For other causes of death, age-adjusted relative risks in men ranged from 2.4 for circulatory diseases to 5.3 for diseases of the nervous system. In women, age-adjusted relative risks ranged from 1.5 for circulatory diseases to 2.5 for diseases of the nervous system (Table 2). For all cause of death categories, the excess mortality was particularly high shortly after the fracture and declined during the subsequent year (Fig. 3).

**Table 2:** One-year mortality (%), all-cause and cause-specific excess mortality in hip fracture patients compared with controls^1^

	Men			Women		
	Observed one-year deaths in hip fracture patients (%)	Observed one-year deaths in controls (%)^1^	HR (95% CI)^2^	Observed one-year deaths in hip fracture patients (%)	Observed one-year deaths in controls (%)^1^	HR (95% CI)^2^
Total deaths	31.7	9.3	4.1 (3.9, 4.2)	20.4	8.8	2.4 (2.3, 2.4)
External causes	8.6	0.2	48 (41, 56)	5.2	0.2	34 (30, 38)
Circulatory diseases	7.4	3.7	2.4 (2.3, 2.5)	5.7	3.8	1.5 (1.5, 1.6)
Mental/behavioural	1.5	0.4	5.1 (4.5, 5.9)	1.2	0.6	2.2 (2.0, 2.4)
Neoplasms	5.7	2.2	3.1 (2.9, 3.3)	2.9	1.5	2.1 (2.0, 3.3)
Nervous system	1.1	0.3	5.3 (4.6, 6.3)	0.8	0.3	2.5 (2.2, 2.8)
Respiratory diseases	3.5	1.2	3.7 (3.5, 4.1)	1.6	0.9	1.8 (1.7, 2.0
Other causes	3.9	1.3	3.5 (3.3, 3.8)	2.9	1.6	1.9 (1.8, 2.0)

^1^*Controls* refer to age- and sex matched individuals drawn from the same cohort as the hip fracture patients: Individuals 50 years and older identified in the Norwegian Population and Housing Census 2001, conditioned on being alive, resident in Norway and free of hip fracture on the date of the patient’s hip fracture (index date), and followed with regard to deaths

^2^Age-adjusted hazard ratios (HR) with 95% confidence intervals (CI) in hip fracture patients vs. controls

## Discussion

In this nationwide study in the Norwegian population, there was a high excess mortality shortly after hip fracture, decreasing through the year after the fracture. The excess mortality was higher in men compared with women, as shown in many populations [1, 3], and we also found this sex difference to be true across all major cause of death categories and thus not limited to mechanisms associated with certain underlying causes of death. For the majority (53.8%) of early deaths (within 30 days) after hip fracture, the accident involving the injury was identified as the underlying cause of death, defined as the event that initiated the train of morbid events leading directly to death. This underlying cause of death dominated through the subsequent months, and accounted for approximately one-fourth of deaths by one year after the hip fracture. Patients with a subsequent hip fracture comprised a minority of the deaths from external causes after a hip fracture, even in the medium term (6–12 months). By one year post hip fracture, external causes and circulatory diseases comprised a similar proportion of deaths. These were followed by (in descending order) neoplasms, respiratory diseases, mental and behavioural disorders, and diseases of the nervous system and the sense organs. When compared with the age- and sex-matched population without hip fracture, the one-year excess mortality associated with an external cause of death (usually the accidental fall involving the fracture), was, not surprisingly, very high. It is conceivable that this high excess mortality could be reduced by strengthening the efforts for follow-up and care with early mobilization and rehabilitation to prevent life-threatening complications after hip fracture. However, the risk of dying from all other major cause of death categories was also clearly increased during the first year after the hip fracture compared with controls, and particularly so during the first weeks and months. While some recorded causes of death in hip fracture patients can be seen as typical complications of the injury (e.g., pulmonary embolisms), others are unrelated to the injury (e.g., neoplasms). For others again, it cannot be determined with confidence whether they are complications to the injury or whether they represent general comorbidity and frailty in the patient.

Inferring the case fatality of hip fractures based on cause of death statistics will necessarily be hampered by imprecision due to unclear or undocumented causal trajectories. The quality of the data depends on the level of detail on the death certificate, often based on insufficient information about the deceased. In addition, the underlying cause of death is dictated by uniform rules for interrelationships between the diagnoses recorded on the death certificate and may not necessarily represent the reality of events in all individuals. Unintended variation may occur due to unawareness of the consequences of these rules. Based on the ICD-10 coding rules, an accident causing a fracture injury will be accepted as underlying cause of death if a medical condition that may plausibly represent a complication following a fracture is listed as the more immediate cause. In contrast, e.g. a cancer diagnosis will not be accepted as a complication following an accidental fracture, and in such cases, the cancer diagnosis will be defined as the underlying cause of death while the fracture diagnosis is discarded [12]. A substantial proportion of those who died during the first year after hip fracture had an underlying cause of death directly related to the injury (e.g., an accidental fall), or with missing information about circumstances (X59; exposure to unspecified factor). A hip fracture injury is rarely immediately fatal but may be followed by complications such as e.g., myocardial infarction, deep vein thrombosis with pulmonary embolism, pneumonia, urinary tract or other infections, that may in some instances be life-threatening. While completing the death certificate, certifying physicians may sometimes be most attentive to the final complications (immediate cause of death) and may unwittingly omit the accidental injury that started the chain of events, potentially underestimating the role of the hip fracture in the patient’s death. On the other hand, patients who suffer a hip fracture are often frailer and more comorbid than their age- and sex-matched non-fractured counterparts [26] and thus at greater risk of both sustaining a fracture and of dying from pre-existing illness. In that sense, the hip fracture may be viewed as an indicator of multimorbidity, and the causal role of the hip fracture in the patient’s death may sometimes be overestimated when classified as underlying cause of death according to the coding rules. With increasing time since the hip fracture occurred, the likelihood increases that the fracture will be overlooked when completing a death certificate. While we cannot exclude the possibility that this could partly contribute to the declining proportion of injury deaths by time since hip fracture, its contribution cannot be quantified. Although early excess mortality following a hip fracture is particularly high, we have previously shown that excess all-cause mortality persists for more than ten years [3]. We found that with increasing time after the fracture, the distribution of causes of deaths converged towards that in the general background population [27].

Similar patterns of causes of death after hip fracture have previously been reported in nationwide data from Denmark [28]. Accidents represented the most common cause of one-year deaths in patients with hip fracture during 1981–2001, accounting for 32.8% to 38.5% depending on calendar years. These were followed by deaths from cardiovascular disease, cancer and cerebrovascular disease [28]. In a more recent analysis also using nationwide registry data from Denmark, short-term causes of death (30 days) were described in patients with a hip fracture during 2002–2012 [29], corresponding to the time frame of our study. In that study, ‘unspecified cause related to the hip fracture’ accounted for approximately one-third (32.3%) of 30-day mortality after hip fracture, thus representing a lower proportion of the 30-day deaths than in our data, while the proportion of deaths from cardiovascular disease in the Danish study corresponded to that in our study, accounting for approximately one-fifth (21.1%) of 30-day mortality.

Excess post-hip fracture mortality from all major causes of death has also been documented in other countries. A study from Sweden with a smaller sample size (1,013 hip fracture patients and 2,026 matched controls) showed one-year excess mortality from the three most common causes of death; cardiovascular disease, cancer, and pneumonia [30]. For these outcomes, one-year mortality was in the magnitude of 3 to 4 times higher than in the background population, comparable to our study. In a Finnish study of 428 hip fracture patients, excess mortality was also observed in all major cause of death categories [31]. Annual relative mortality ranged from 2.5 for deaths from neoplasms to 8.4 for deaths from diseases of the digestive system. In a population-based matched cohort from Korea including 3,383 patients who suffered a hip fracture 2003–2012 [32], overall all-cause mortality was two-fold higher in hip fracture patients compared with controls during a follow-up of up to 11 years (mean 4.5 years), with higher mortality from all major cause of death categories except for mental and behavioural disorders [32]. The cohort study from Korea did not consider trends in cause of death patterns according to time since the fracture. Although between-country variations in certifying and coding practices complicate direct comparisons, it seems to be a general observation that hip fractures entail increased mortality from several major causes of death.

Our study included nationwide registry data covering the total population of Norway over two decades. Norway has universal public healthcare and hip fracture surgeries are performed in public hospitals. The Norwegian Cause of Death Registry has a uniform coding system in line with international rules proposed by the World Health Organization, facilitating comparisons with other countries. Yet, the available information relies on diagnoses registered on the death certificates, completed by the attending physician. To ensure high accuracy of the registered causes of death, there have been efforts to return incomplete death certificates to the certifying physicians. This is particularly important when deaths have been assigned ICD-10 codes that are uninformative for identifying the true underlying cause of death, often referred to as ‘garbage codes’, of which common examples are heart failure and sudden death [33–36]. Since implementing ACME in 2005, the garbage code X59.0 ‘Exposure to unspecified factor causing fracture’ is assigned if a hip fracture is recorded with missing external cause. During 2015–2016, a quality assurance project was carried out for deaths coded with X59 [37]. Based on predictors such as sex, age, place of death and nature of the injury, it was estimated that 97% of deaths coded with ICD-10 X59 during 2005–2014 could be redistributed to an accidental fall [36, 37]. More recently, electronic death certificates have been gradually implemented in Norway and are mandatory from 2022, using a digital system that assists the certifying physicians in submitting the necessary information in a standardized way, thereby aiming to obtain improved cause of death statistics in the future [10].

In summary, a hip fracture may be considered an indicator of deteriorating health and multimorbidity with ageing, or an adverse life event that triggers acceleration of ageing with accompanying medical complications, exacerbation of chronic diseases, and decline in physical and cognitive function. Our findings of a particularly high short-term excess mortality from the injury as well as an excess risk of death from all causes during the first year after a hip fracture supports both these notions.

## Conclusions

The traumatic injury of a hip fracture is the leading underlying cause of death among older patients who survive less than one year after their fracture. This finding underscores the importance of preventing falls, and thus fractures, among older adults. While the excess mortality from accidental falls leading to hip fractures is very high, hip fracture patients have excess one-year mortality from all major causes of death.

## Data Availability

Corresponding Author K.H. may be contacted for information about the data and materials. The data that support the findings of this study are available upon application to the respective data owners (Norwegian Institute of Public Health, Norwegian Directorate of Health, and Statistics Norway) but restrictions apply to the availability of these data, which were used under approval for the purpose of the current research project, and so are not publicly available.

## Abbreviations

ACME: Automated Classification of Medical Entities
ICD-10: International Classification of Diseases, 10^th^ edition
NOREPOS: The Norwegian Epidemiologic Osteoporosis Studies
NORHip: The Norwegian Epidemiologic Osteoporosis Studies (NOREPOS) hip fracture database

## References

[1] Haentjens P, Magaziner J, Colon-Emeric CS, Vanderschueren D, Milisen K, Velkeniers B, Boonen S (2010). Meta-analysis: excess mortality after hip fracture among older women and men. Ann Intern Med.

[2] Katsoulis M, Benetou V, Karapetyan T, Feskanich D, Grodstein F, Pettersson-Kymmer U, Eriksson S, Wilsgaard T, Jørgensen L, Ahmed LA (2017). Excess mortality after hip fracture in elderly persons from Europe and the USA: the CHANCES project. J Intern Med.

[3] Omsland TK, Emaus N, Tell GS, Magnus JH, Ahmed LA, Holvik K, Center J, Forsmo S, Gjesdal CG, Schei B (2014). Mortality following the first hip fracture in Norwegian women and men (1999-2008). A NOREPOS study. Bone..

[4] Ranhoff AH, Holvik K, Martinsen MI, Domaas K, Solheim LF (2010). Older hip fracture patients: three groups with different needs. BMC Geriatr.

[5] Søgaard AJ, Meyer HE, Emaus N, Grimnes G, Gjesdal CG, Forsmo S, Schei B, Tell GS (2014). Cohort profile: Norwegian Epidemiologic Osteoporosis Studies (NOREPOS). Scand J Public Health.

[6] Omsland TK, Holvik K, Meyer HE, Center JR, Emaus N, Tell GS, Schei B, Tverdal A, Gjesdal CG, Grimnes G (2012). Hip fractures in Norway 1999–2008: time trends in total incidence and second hip fracture rates: a NOREPOS study. Eur J Epidemiol.

[7] Søgaard AJ, Holvik K, Meyer HE, Tell GS, Gjesdal CG, Emaus N, Grimnes G, Schei B, Forsmo S, Omsland TK (2016). Continued decline in hip fracture incidence in Norway: a NOREPOS study. Osteoporos Int.

[8] Kjeldgaard HK, Meyer HE, O'Flaherty M, Apalset EM, Dahl C, Emaus N, Fenstad AM, Furnes O, Gjertsen JE, Hoff M (2022). Impact of total hip replacements on the incidence of hip fractures in Norway during 1999–2019. A NOREPOS study. J Bone Miner Res..

[9] Method for collection and quality assurance of data for the NOREPOS Hip Fracture Database. www.norepos.no/documentation.

[10] Norwegian Cause of Death Registry. https://www.fhi.no/en/hn/health-registries/cause-of-death-registry/.

[11] Pedersen AG, Ellingsen CL (2015). Data quality in the Causes of Death Registry. J Norw Med Assoc (Tidsskr Nor Laegeforen)..

[12] World Health Organization. International Statistical Classification of Diseases and Related Health Problems, 10th Revision (ICD-10) Volume 2, Instruction Manual. Geneva: World Health Organization, 2010. Fifth edition, 2016. https://icd.who.int/browse10/Content/statichtml/ICD10Volume2_en_2019.pdf.

[13] Johansson LA, Westerling R (2002). Comparing hospital discharge records with death certificates: can the differences be explained?. J Epidemiol Community Health.

[14] Lu TH (2003). Using ACME (Automatic Classification of Medical Entry) software to monitor and improve the quality of cause of death statistics. J Epidemiol Community Health.

[15] Lu TH, Tsau SM, Wu TC (2005). The Automated Classification of Medical Entities (ACME) system objectively assessed the appropriateness of underlying cause-of-death certification and assignment. J Clin Epidemiol.

[16] Iris software. https://www.bfarm.de/EN/Code-systems/Collaboration-and-projects/Iris-Institute/_node.html.

[17] European Shortlist for Causes of Death. https://ec.europa.eu/eurostat/ramon/nomenclatures/index.cfm?TargetUrl=LST_NOM_DTL&StrNom=COD_2012.

[18] R Development Core Team. R: A language and environment for statistical computing. R Foundation for Statistical Computing, Vienna, Austria. https://www.R-project.org/.

[19] StataCorp. Stata statistical software: release 17. College Station: StataCorp LLC; 2021.

[20] Ohneberg K, Beyersmann J, Schumacher M (2019). Exposure density sampling: dynamic matching with respect to a time-dependent exposure. Stat Med.

[21] Ohneberg K (2019). Sampling Designs for Complex Time-to-Event Data in Clinical and Epidemiological Studies.

[22] Carstensen B, Plummer M, Laara E, Hills M. Epi: A Package for Statistical Analysis in Epidemiology. R package version 2.44. https://CRAN.R-project.org/package=Epi.

[23] Grambsch PM, Therneau TM (1994). Proportional hazards tests and diagnostics based on weighted residuals. Biometrika.

[24] Royston P, Lambert PC. Flexible parametric survival analysis using Stata: beyond the Cox Model. College Station: Stata Press; 2011.

[25] Sjölander A, Greenland S (2013). Ignoring the matching variables in cohort studies - when is it valid and why?. Stat Med.

[26] Holvik K, Hjellvik V, Karlstad Ø, Gunnes N, Hoff M, Tell GS, Meyer HE (2022). Contribution of an extensive medication-based comorbidity index (Rx-Risk) in explaining the excess mortality after hip fracture in older Norwegians: a NOREPOS cohort study. BMJ Open.

[27] Bævre K. Life expectancy in Norway. In: The Norwegian Public Health Report. Oslo: Norwegian Institute of Public Health; 2018. https://www.fhi.no/en/op/hin/population/life-expectancy/.

[28] Vestergaard P, Rejnmark L, Mosekilde L (2007). Has mortality after a hip fracture increased?. J A, Geriatr Soc.

[29] Rohold CK, Lauritzen JB, Jørgensen HL (2022). Causes of death among 93.637 hip fracture patients- data based on the Danish national registry of causes of death. Eur J Trauma Emerg Surg..

[30] von Friesendorff M, McGuigan FE, Wizert A, Rogmark C, Holmberg AH, Woolf AD, Åkesson K (2016). Hip fracture, mortality risk, and cause of death over two decades. Osteoporos Int.

[31] Panula J, Pihlajamäki H, Mattila VM, Jaatinen P, Vahlberg T, Aarnio P, Kivelä SL (2011). Mortality and cause of death in hip fracture patients aged 65 or older: a population-based study. BMC Musculoskelet Disord.

[32] Choi HG, Lee YB, Rhyu SH, Kwon BC, Lee JK (2018). Mortality and cause of death postoperatively in patients with a hip fracture: a national cohort longitudinal follow-up study. Bone Joint J.

[33] Naghavi M, Makela S, Foreman K, O'Brien J, Pourmalek F, Lozano R (2010). Algorithms for enhancing public health utility of national causes-of-death data. Popul Health Metr.

[34] Naghavi M, Richards N, Chowdhury H, Eynstone-Hinkins J, Franca E, Hegnauer M, Khosravi A, Moran L, Mikkelsen L, Lopez AD (2020). Improving the quality of cause of death data for public health policy: are all 'garbage' codes equally problematic?. BMC Med.

[35] Mikkelson L, Richards N, Lopez AD. Redefining ‘garbage codes’ for public health policy: Report on the expert group meeting, 27–28 February 2017. Civil Registration and Vital Statistics (CRVS) best-practice and advocacy. Melbourne: University of Melbourne; 2019.

[36] Ellingsen CL, Alfsen GC, Ebbing M, Pedersen AG, Sulo G, Vollset SE, Braut GS (2022). Garbage codes in the Norwegian cause of death registry 1996–2019. BMC Public Health.

[37] Ellingsen CL, Ebbing M, Alfsen GC, Vollset SE (2018). Injury death certificates without specification of the circumstances leading to the fatal injury - the Norwegian cause of death registry 2005–2014. Popul Health Metr.

