# Correction to: Cause-specific excess mortality after hip fracture: the Norwegian Epidemiologic Osteoporosis Studies (NOREPOS)

**DOI:** 10.1186/s12877-023-04013-x

**Published:** 2023-05-16

**Authors:** Kristin Holvik, Christian Lycke Ellingsen, Siri Marie Solbakken, Trine Elisabeth Finnes, Ove Talsnes, Guri Grimnes, Grethe S. Tell, Anne-Johanne Søgaard, Haakon E. Meyer

**Affiliations:** 1 Department of Physical Health and Ageing, Norwegian Institute of Public Health, P. O. Box 222, Skøyen, Oslo 0213 Norway; 2 Department of Pathology, Stavanger University Hospital, Stavanger, Norway; 3 Department of Global Public Health and Primary Care, University of Bergen, Bergen, Norway; 4 Department of Endocrinology, Innlandet Hospital Trust, Hamar, Norway; 5 Department of Endocrinology, Oslo University Hospital, Oslo, Norway; 6 Department of Orthopedics, Innlandet Hospital Trust, Elverum, Norway; 7 Department of Clinical Medicine, UiT The Arctic University of Norway, Tromsø, Norway; 8 Division of Internal Medicine, University Hospital of North Norway, Tromsø, Norway; 9 Department of Community Medicine and Global Health, University of Oslo, Oslo, Norway

Following publication of the original article [1], we have been informed that an error has occurred in the caption of Fig. 3.

Originally published phrase:

From top left: All-cause deaths; deaths from external causes; deaths from neoplasms; deaths from diseases of the nervous system and the sense organs. From bottom left: Deaths from circulatory diseases; deaths from mental and behavioural disorders; deaths from diseases of the respiratory system; deaths from other causes.

Corrected caption phrase:

Panel a: all-cause deaths; Panel b: deaths from external causes; Panel c: deaths from neoplasms; Panel d: deaths from diseases of the nervous system and the sense organs; Panel e: deaths from circulatory diseases; Panel f: deaths from mental and behavioural disorders; Panel g: deaths from diseases of the respiratory system; Panel h: Deaths from other causes.

The original article has been corrected.
